# First-principles and cluster expansion investigation of Ti–V–Mn AB_2_ alloys: stability, electronic structure, and mechanical properties

**DOI:** 10.1007/s00894-026-07016-8

**Published:** 2026-10-23

**Authors:** V. Chauke, D. M. Tshwane, H. R. Chauke

**Affiliations:** 1 Materials Modelling Centre, University of Limpopo, Private Bag X 1106, Sovenga, 0727 South Africa; 2 National Institute for Theoretical and Computational Sciences (NITheCS), Stellenbosch, South Africa; 3 Green Technology Confucius Institute, University of Venda, Thohoyandou, 0950 South Africa

**Keywords:** AB_2_ alloys, Cluster expansion, Density functional theory, Thermodynamic stability, Electronic structure, Mechanical stability

## Abstract

**Context:**

Ti–V–Mn AB_2_-type alloys are promising materials for hydrogen storage applications, but their intrinsic properties are strongly influenced by chemical composition and atomic ordering. The large number of possible atomic configurations makes it challenging to establish how configurational ordering governs the energetic, electronic, and mechanical behaviour of these alloys. In this study, a combined cluster expansion and first-principles approach is used to investigate the relationship between atomic configuration and the energetic, electronic, and mechanical properties of selected ground-state Ti–V–Mn AB_2_ configurations. Among the three selected configurations, Ti_8_V_9_Mn_7_ exhibits the most negative heat of formation, indicating the most favourable formation energetics within the investigated structures. All configurations exhibit metallic character, with states near the Fermi level predominantly derived from Ti, V, and Mn 3d orbitals. The calculated elastic properties indicate mechanical stability for all three structures, while Ti_8_V_8_Mn_8_ exhibits the highest bulk, shear, and Young’s moduli. Ti_4_V_6_Mn_2_ has the highest calculated Pugh ratio and Poisson’s ratio, indicating the greatest predicted ductility among the investigated configurations. These results demonstrate that configurational ordering influences the energetic, electronic, and elastic properties of Ti–V–Mn alloys and provide a computational baseline for subsequent investigations of hydrogen-related behaviour.

**Methods:**

Cluster expansion calculations were performed using the UNCLE code within the MedeA computational environment to explore configurational energetics and construct the ground-state convex hull. Selected ground-state configurations were subsequently investigated using spin-polarised density functional theory as implemented in the Vienna Ab initio Simulation Package (VASP), employing the GGA-PBE exchange–correlation functional and projector augmented-wave potentials with a plane-wave basis. Structural optimisation, partial and total densities of states, and elastic constants were calculated. Brillouin zone sampling used a gamma-centred k-point mesh with a k-spacing of 0.5 Å⁻^1^, while first-order Methfessel–Paxton smearing with a width of 0.2 eV was applied. Bulk, shear, and Young’s moduli were obtained using the Voigt–Reuss–Hill approximation, and Pugh ratios and Poisson’s ratios were used to assess the predicted ductile or brittle behaviour.

## Introduction

Ti-based transition metal alloys have attracted considerable attention due to their excellent combination of structural stability, mechanical performance, and tenable thermodynamic properties [1, 2]. Ti-based alloys are widely explored in structural and functional applications due to their favourable strength-to-weight ratio, corrosion resistance, phase stability, and adaptable alloy chemistry [3]. Among these materials, AB_2_-type Ti–V–Mn-based alloys are particularly interesting due to their favourable phase stability, adjustable chemical composition, and ability to accommodate different elemental substitutions [4, 5]. The properties of these alloys are strongly influenced by atomic arrangement, compositional variation, and interactions between constituent transition metal elements [6]. Understanding the relationship between atomic configuration and intrinsic material properties is therefore essential for optimising Ti–V–Mn-based alloys for advanced functional applications, including solid-state hydrogen storage [7].

Ti–V–Mn alloy systems have attracted increasing interest due to the complementary effects of their constituent elements on phase stability, electronic structure, and mechanical behaviour. The combination of Ti, V, and Mn provides a flexible alloying platform in which compositional variation can be used to modify phase stability, lattice characteristics, and mechanical behaviour. In particular, V and Mn additions influence the atomic arrangement and bonding environment within Ti-based alloys, making the control of chemical ordering essential for tailoring their structural and functional properties [8]. Variations in atomic arrangement can significantly influence electronic structure, bonding characteristics, thermodynamic stability, and elastic properties of Ti–V–Mn alloys [9, 10]. Therefore, establishing a clear relationship between configurational ordering and intrinsic material properties is critical for understanding the stability and mechanical response of these alloy systems [11].

Recent first-principles studies of Ti-based Laves phases have demonstrated that chemical composition and atomic arrangement influence their structural, electronic, and mechanical properties. In particular, investigations of TiCr_2_, TiMn_2_, and TiFe_2_ C14 Laves phases have shown that changes in chemical composition are reflected in their elastic response, electronic structure, and bonding characteristics [12]. Related calculations on TiCrMn further demonstrated the influence of chemical substitution on structural stability, elastic properties, electronic structure, and hydrogen-related behaviour [13]. Similarly, studies on Ti–V-based alloys have shown that changes in V concentration influence structural stability and elastic behaviour due to variations in atomic interactions and electronic structure, highlighting the importance of composition control in tailoring Ti-based alloy properties [14].

Furthermore, Ti–Mn-containing alloy systems have attracted increasing attention due to the ability of Mn to modify phase stability and influence the interaction between neighbouring transition metal atoms. First-principles investigations of TiMn_2_ and related C14 Laves phases have reported composition- and structure-dependent variations in their stability, electronic structure, and elastic properties [12, 15]. Therefore, understanding the relationship between atomic configuration and intrinsic material properties is essential for guiding the rational design of Ti–V–Mn alloys with improved structural stability and functional performance.

Previous first-principles investigations of Ti-based C14 Laves phases have established the importance of crystal structure, elastic response, and electronic interactions in determining their stability and mechanical behaviour. Nong et al. investigated TiX_2_ (X = Cr, Mn, Fe) C14 Laves phases and reported their structural, elastic, and electronic properties, including the mechanical stability of the investigated compounds [12]. In a related study, TiCrMn was examined with particular emphasis on structural stability, elastic properties, electronic structure, and hydrogen storage behaviour [13]. These studies provide an important reference framework for assessing the structural, electronic, and mechanical characteristics of the Ti–V–Mn configurations investigated in the present work.

The relevance of these properties is particularly important for Ti–V–Mn alloys considered for hydrogen storage applications, where structural stability and the response of the host alloy to hydrogen incorporation are closely related to the underlying chemical bonding and mechanical behaviour [4, 16]. Variations in composition and atomic configuration can modify the local chemical environment and, consequently, the intrinsic stability and mechanical response of the alloy. Establishing these properties therefore provides a necessary materials-level basis for assessing Ti–V–Mn alloys as hydrogen storage materials [4, 16]. In the present work, the focus is placed on the configurational stability, electronic structure, and intrinsic mechanical properties of selected Ti–V–Mn configurations, providing a foundation for subsequent investigations of their hydrogen–alloy interactions.

Despite the importance of understanding configurational effects in Ti–V–Mn alloys, their multicomponent nature introduces significant computational challenges due to the large number of possible atomic arrangements. Although density functional theory (DFT) provides reliable predictions of structural, electronic, and mechanical properties, direct evaluation of all possible atomic configurations becomes computationally demanding as alloy complexity increases [17]. To address this limitation, the cluster expansion (CE) method has emerged as a powerful approach for modelling configurational thermodynamics in multicomponent alloys. The CE formalism maps the configurational energy of an alloy onto a set of effective cluster interactions, allowing efficient exploration of large configurational spaces while maintaining first-principles-level accuracy [18]. This method has been successfully applied to predict phase stability, ordering behaviour, and thermodynamic properties in complex alloy systems [19].

Previous studies combining cluster expansion with first-principles calculations have successfully investigated structure–property relationships in complex alloy systems, providing insights into the influence of atomic configuration on phase stability, electronic structure, and mechanical behaviour [20]. Although Ti–V–Mn-based alloys have received considerable attention due to their promising functional properties, the influence of configurational ordering on the coupled thermodynamic, electronic, and mechanical behaviour of CE-derived ground-state structures remains insufficiently understood. Limited studies have systematically linked convex hull–derived atomic configurations with electronic structure, elastic stability, and bonding characteristics in Ti–V–Mn AB_2_-type systems. Establishing these relationships is important for understanding how atomic-scale configurations are associated with the intrinsic stability and mechanical properties of hydrogen storage alloys.

In this study, a combined cluster expansion and first-principles approach is used to investigate the relationship between atomic configuration and the energetic, electronic, and mechanical properties of Ti–V–Mn AB_2_ alloys. The cluster expansion method is employed to explore the configurational space and identify energetically stable configurations from the convex hull. Three representative configurations, namely Ti_4_V_6_Mn_2_, Ti_8_V_9_Mn_7_, and Ti_8_V_8_Mn_8_, are subsequently selected from the predicted ground-state structures for detailed density functional theory calculations due to their favourable thermodynamic stability and distinct compositional characteristics. Their energetic stability, electronic density of states, and mechanical properties are systematically analysed and compared. This work provides insight into the relationship between atomic configuration and the stability and intrinsic properties of Ti–V–Mn alloys, establishing a computational foundation for future optimisation of AB_2_-type alloy systems.

## Computational method

### Cluster expansion calculations

The cluster expansion (CE) method was carried out to determine the ground-state structures of the Ti–V–Mn alloy system employing the UNCLE code as implemented in the MedeA computing environment [19, 21]. The CE was constructed using the (P6_3/mmc) parent lattice, corresponding to the C14-type AB_2_ Laves-phase framework. In this model, Ti occupies the inactive sublattice, while V and Mn occupy the active sublattice and are allowed to interchange configurationally. The CE approach was used to explore the configurational space of the alloy system by generating different atomic arrangements and evaluating their configurational energetics.

During the fitting procedure, the CE calculations were optimised using a genetic algorithm in which different structures were iteratively added to the training set until convergence was achieved. The fitting process was allowed to proceed for a maximum of 40 iterations, adding up to 20 new structures per iteration, beginning from an initial training set of 20 structures.

The predictive accuracy of the CE was evaluated using the cross-validation score (CVS). A CE model is considered reliable when the CVS is below 5 meV per active atom position [1]. In the present study, the final CE model achieved a CVS value of approximately 2.8–2.9 meV/pos., indicating excellent agreement between the CE-predicted energies and the DFT-calculated energies. The thermodynamically stable configurations were identified from the DFT ground-state line constructed from the convex hull of formation energies.

### Density functional theory calculations

The ground-state structures predicted by the cluster expansion calculations were fully optimised using DFT as implemented in the Vienna Ab initio Simulation Package (VASP) [22, 23]. The generalised gradient approximation (GGA) within the Perdew–Burke–Ernzerhof (PBE) functional was employed to describe the exchange–correlation interactions [24]. The electron–ion interactions were treated using the projector augmented wave (PAW) method [25]. Based on the plane-wave cut-off convergence tests, cut-off energies of 350 eV for Ti_8_V_9_Mn_7_ and Ti_8_V_8_Mn_8_, and 400 eV for Ti_4_V_6_Mn_2_, were adopted for the respective calculations. Structural optimisation was carried out using the conjugate-gradient algorithm by allowing both the atomic positions and lattice parameters to relax. The electronic self-consistency convergence criterion was set to $1\times {10}^{-7}$ eV, while the ionic relaxation was performed until the Hellmann–Feynman forces on each atom were less than 0.02 eV/Å. Gamma-centred k-point meshes of 7 × 5 × 4, 15 × 9 × 15, and 6 × 4 × 4 were employed for Ti_8_V_9_Mn_7_, Ti_4_V_6_Mn_2_, and Ti_8_V_8_Mn_8_, respectively, for Brillouin zone integration. These correspond to k-point spacings of approximately 0.181 × 0.161 × 0.184 Å⁻^1^, 0.095 × 0.092 × 0.095 Å⁻^1^, and 0.215 × 0.184 × 0.200 Å⁻^1^, respectively. The first-order Methfessel–Paxton smearing method with a width of 0.2 eV was used during the calculations [26]. Spin-polarised calculations were performed for all structures to properly account for the magnetic contributions of the transition metal elements. Based on the CE ground-state diagram, three representative configurations, namely Ti_4_V_6_Mn_2_, Ti_8_V_9_Mn_7_, and Ti_8_V_8_Mn_8_, were selected for detailed first-principles investigation due to their relatively low formation energies and thermodynamic stability.

## Results and discussion

### Ground-state structures and configurational stability

**Fig. 1:**
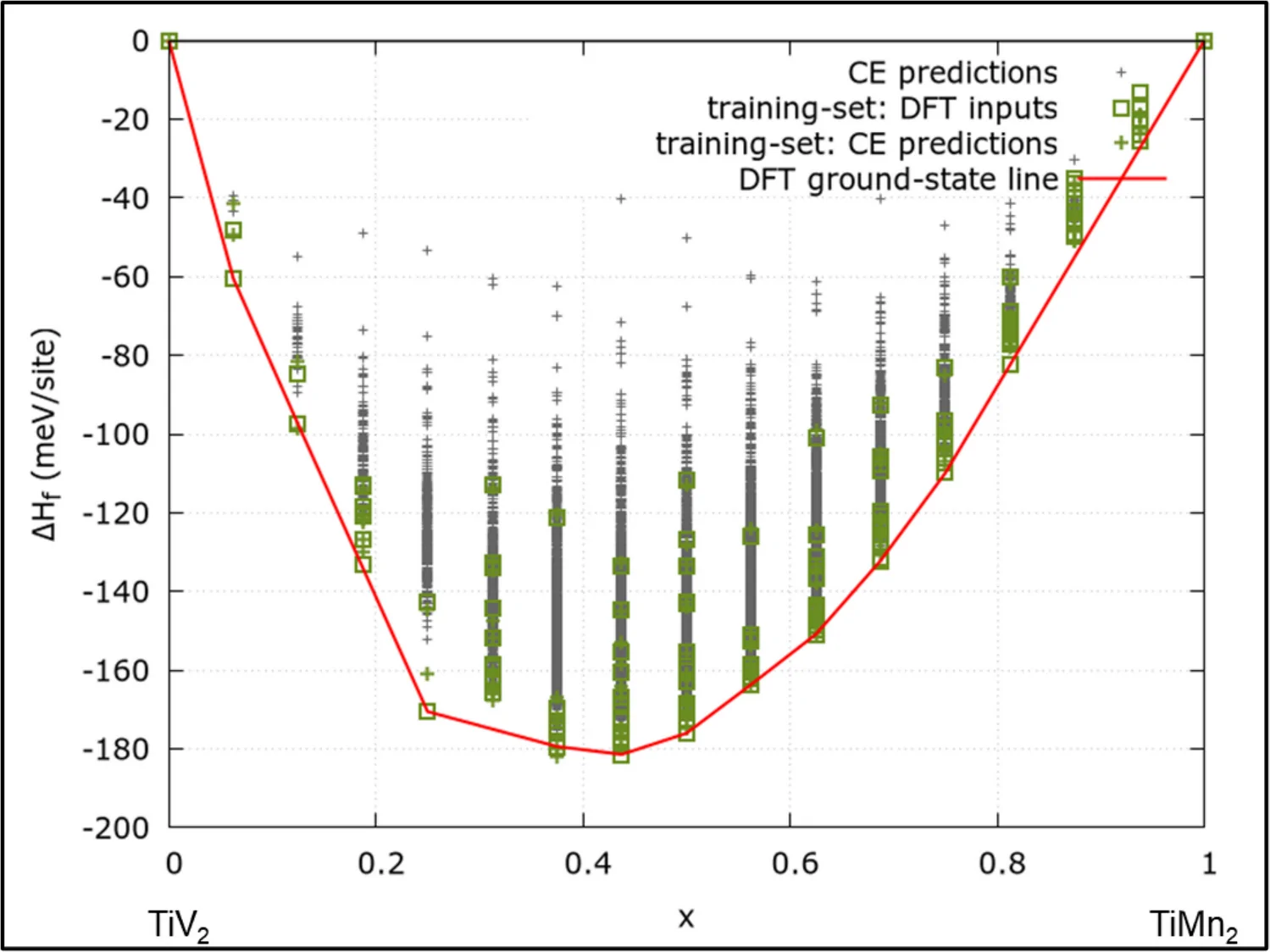
Ground-state convex hull of the Ti–V–Mn alloy system obtained from cluster expansion calculations. The composition coordinate *x* represents the Mn fraction on the active V/Mn B-sublattice, *x* = N_Mn_/(N_V_ + N_Mn_)

The configurational stability of the Ti–V–Mn alloy system was investigated using the cluster expansion technique, and the resulting ground-state diagram is presented in Fig. 1. The convex hull exhibits a pronounced stabilisation across the intermediate composition region, where the formation energies become increasingly negative relative to the end-member compositions. The negative formation energies observed across the intermediate composition range indicate that configurational ordering lowers the total energy of the alloy system and stabilises the formation of ordered Ti–V–Mn AB_2_-type Laves-derived phases, originating from the TiV_2_-TiMn_2_ binary end-member structures.

The formation energies shown in Fig. 1 are the cluster expansion formation energies reported by UNCLE per active atom position. In the present cluster expansion model, the V/Mn sites constitute the active sublattice, while Ti occupies the inactive sublattice. Accordingly, the energy scale in Fig. 1 is expressed in meV per active site.

Here, *x* denotes the Mn fraction on the active V/Mn B-sublattice, defined as *x* = N_Mn_/(N_V_ + N_Mn_), with *x* = 0 corresponding to the V-rich end member and *x* = 1 to the Mn-rich end member. The ground-state line contains discrete configurations at specific values of *x*. The lowest formation energy among the calculated ground-state configurations occurs at *x* = 0.438, corresponding to Ti_8_V_9_Mn_7_. The neighbouring ground-state configurations at *x* = 0.375 and *x* = 0.500 also exhibit relatively low formation energies, indicating a low-energy region centred around intermediate V/Mn compositions. Within the intermediate composition region (*x*≈0.4–0.5), the formation energies reach their minimum values, suggesting that configurational ordering between V and Mn atoms significantly lowers the total energy of the system. The deep curvature of the convex hull further implies strong chemical interactions and favourable atomic arrangements within the alloy lattice. The pronounced stabilisation near the intermediate composition range may additionally be associated with reduced local strain energy arising from favourable atomic packing and optimised chemical interactions between V and Mn atoms occupying the B-sublattice. Similar stabilisation behaviour has been reported in cluster expansion–based investigations of multicomponent alloy systems, where favourable atomic ordering was shown to lower configurational energies and promote thermodynamically stable phases [27, 28]. Such behaviour is characteristic of chemically ordered multicomponent transition metal alloy systems.

Moving away from the low-energy intermediate composition region towards the V-rich or Mn-rich ends of the B-sublattice, the formation energies of the ground-state configurations increase. Among the selected configurations, the near-equiatomic Ti_8_V_9_Mn_7_ and Ti_8_V_8_Mn_8_ compositions exhibit enhanced stability compared with the V-rich Ti_4_V_6_Mn_2_ structure, demonstrating the strong influence of compositional balance and atomic ordering on the energetic stability of Ti–V–Mn alloys. The stabilisation observed near the equiatomic compositions may originate from improved electronic interactions and reduced local lattice strain arising from favourable atomic packing [29].

**Fig. 2:**
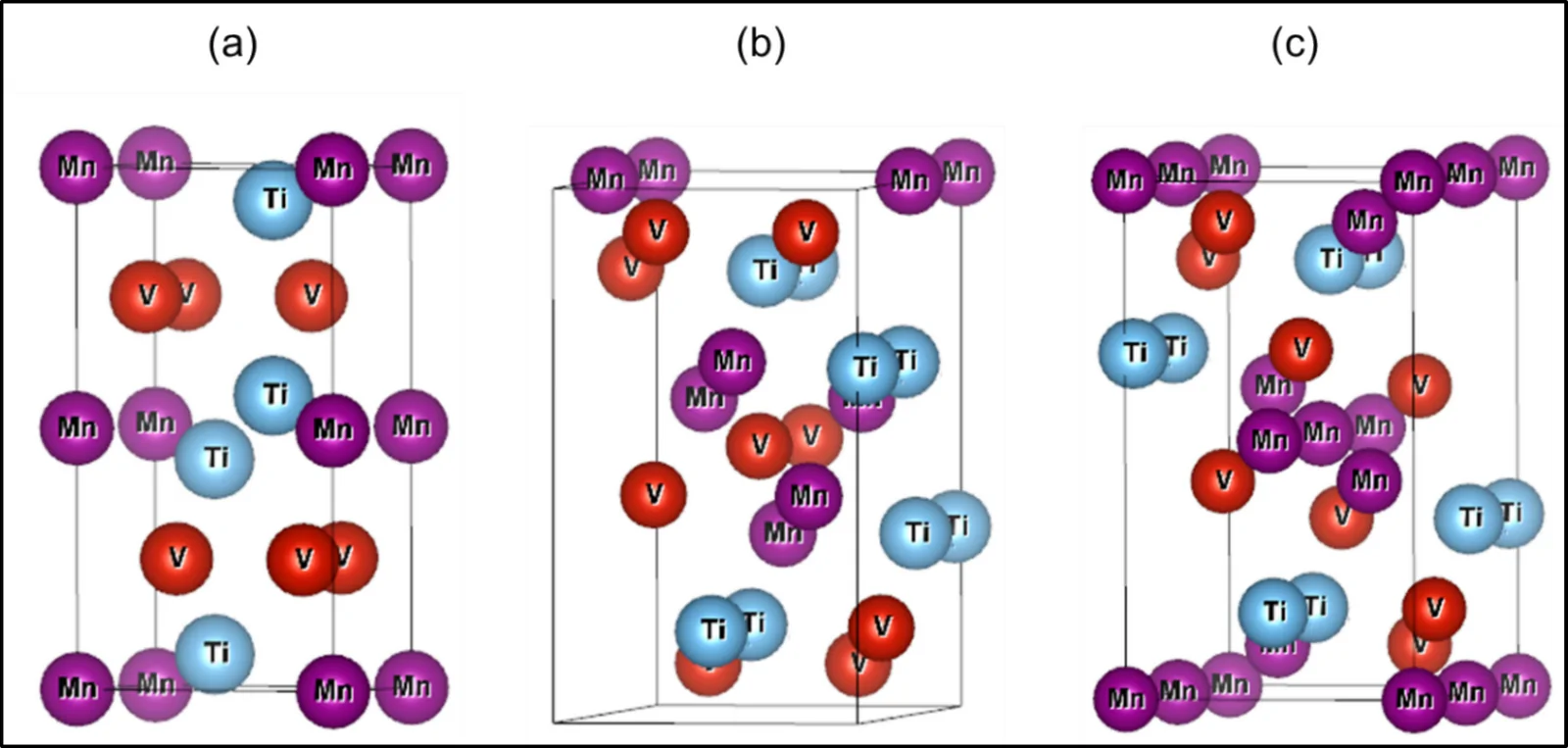
Optimised crystal structures of the selected ground-state Ti–V–Mn configurations: **a** Ti_4_V_6_Mn_2_, **b** Ti_8_V_9_Mn_7_, and **c** Ti_8_V_8_Mn_8_

Three representatives in Fig. 2a–c ground-state structures, namely Ti_4_V_6_Mn_2_, Ti_8_V_9_Mn_7_, and Ti_8_V_8_Mn_8_, respectively, were selected from the convex hull for detailed first-principles investigation due to their relative stability shown by the low formation energies and distinct compositional characteristics. Among the selected structures, Ti_8_V_9_Mn_7_ was found to exhibit the lowest formation energy, indicating enhanced thermodynamic stability compared to the other configurations Ti_4_V_6_Mn_2_ and Ti_8_V_8_Mn_8_. This enhanced stability may be associated with favourable local atomic environments and improved electronic interactions among Ti, V, and Mn atoms.

The optimised ground-state structures shown in Fig. 2 exhibit distinct atomic arrangements and local chemical ordering characteristics that evolve with alloy composition. Ti_4_V_6_Mn_2_ exhibits a relatively ordered structure with strong V-dominated interactions, while Ti_8_V_9_Mn_7_ shows a more homogeneous distribution of Ti, V, and Mn atoms throughout the lattice, consistent with the energetic stabilisation predicted from the convex hull analysis. The Ti_8_V_8_Mn_8_ configuration displays a comparatively symmetric atomic arrangement associated with its near-equiatomic composition, together with increased local atomic disorder. In addition, slight variations in local coordination environments and interatomic spacing among the three structures suggest composition-dependent lattice distortion effects, which are expected to influence the electronic bonding characteristics and mechanical response of the alloy system. Overall, the CE results demonstrate that subtle variations in atomic configuration strongly influence the energetic stability and structural evolution of Ti–V–Mn AB_2_-type alloys.

### Structural parameters and heat of formation

The optimised structural parameters and heat of formation of the investigated binary and ternary Ti–V–Mn configurations are summarised in Table 1. The parent alloys TiV_2_ and TiMn_2_ exhibit characteristic AB_2_-type lattice structures, which serve as reference systems for evaluating the structural evolution of the ternary ground-state configurations. The introduction of Mn into the TiV_2_ framework and the simultaneous mixing of V and Mn atoms within the B-sublattice result in noticeable variations in lattice parameters and unit cell volume, indicating composition-dependent structural distortion effects associated with differences in atomic size and local bonding interactions. The structural parameters and heats of formation of the parent TiV_2_ and TiMn_2_ phases obtained from literature are included as reference values to provide a comparative basis for evaluating the structural and thermodynamic behaviour of the ternary Ti–V–Mn configurations.

**Table 1:** Optimised structural parameters and heats of formation (Δ*H*_*f*_) of the investigated Ti–V–Mn alloy configurations

Structure	Space group	*a* (Å)	*b* (Å)	*c* (Å)	Δ*H*_*f*_ (eV/atom)
TiV_2_	Fd3m	7.124 [30]	7.124 [30]	7.124 [30]	−0.0859 [30]
TiMn_2_	P6_3/mmc	5.090 [31]	5.090	7.733	−0.272 [31]
Ti_4_V_6_Mn_2_	P6_3/mmc	5.092	5.092	7.604	−0.621
Ti_8_V_9_Mn_7_	Pm	4.967	7.811	8.530	−0.757
Ti_8_V_8_Mn_8_	Pnma	4.887	8.552	7.862	−0.705

The Ti_4_V_6_Mn_2_ structure was obtained using a non-standard orientation of the hexagonal unit cell. To maintain consistency with the conventional P6_3/mmc setting, the lattice parameters are reported in Table 1 with *a* = *b* as the equivalent basal-plane parameters and c as the unique hexagonal axis. This representation corresponds to the same relaxed structure and does not involve a change in the calculated atomic configuration or total energy.

The results show that Ti_8_V_9_Mn_7_ exhibits the lowest calculated heat of formation, $\Delta {H}_{f}=-0.757$ eV/atom, followed by Ti_8_V_8_Mn_8_ (-0.705 eV/atom) and Ti_4_V_6_Mn_2_ (-0.621 eV/atom). The difference in $\Delta {H}_{f}$ between Ti_8_V_9_Mn_7_ and Ti_8_V_8_Mn_8_ is 0.052 eV/atom. These results indicate differences in the calculated thermodynamic stability among the investigated Ti–V–Mn configurations. The Ti_8_V_8_Mn_8_ configuration, which is close to the equiatomic composition, exhibits a slightly higher heat of formation than Ti_8_V_9_Mn_7_, whereas Ti_4_V_6_Mn_2_ shows a comparatively higher value. The calculated differences in heat of formation demonstrate that the investigated compositions have distinct energetic stabilities within the considered AB_2_ lattice.

Furthermore, the symmetry reduction observed from the parent binary phases towards the ternary ground-state configurations indicates that chemical substitution and atomic ordering induce local lattice distortion within the AB_2_ framework. Such distortion is associated with differences in the local coordination environments and interatomic bonding topology, which are reflected in the calculated electronic structure and elastic response of the alloy system.

### Electronic properties

**Fig. 3:**
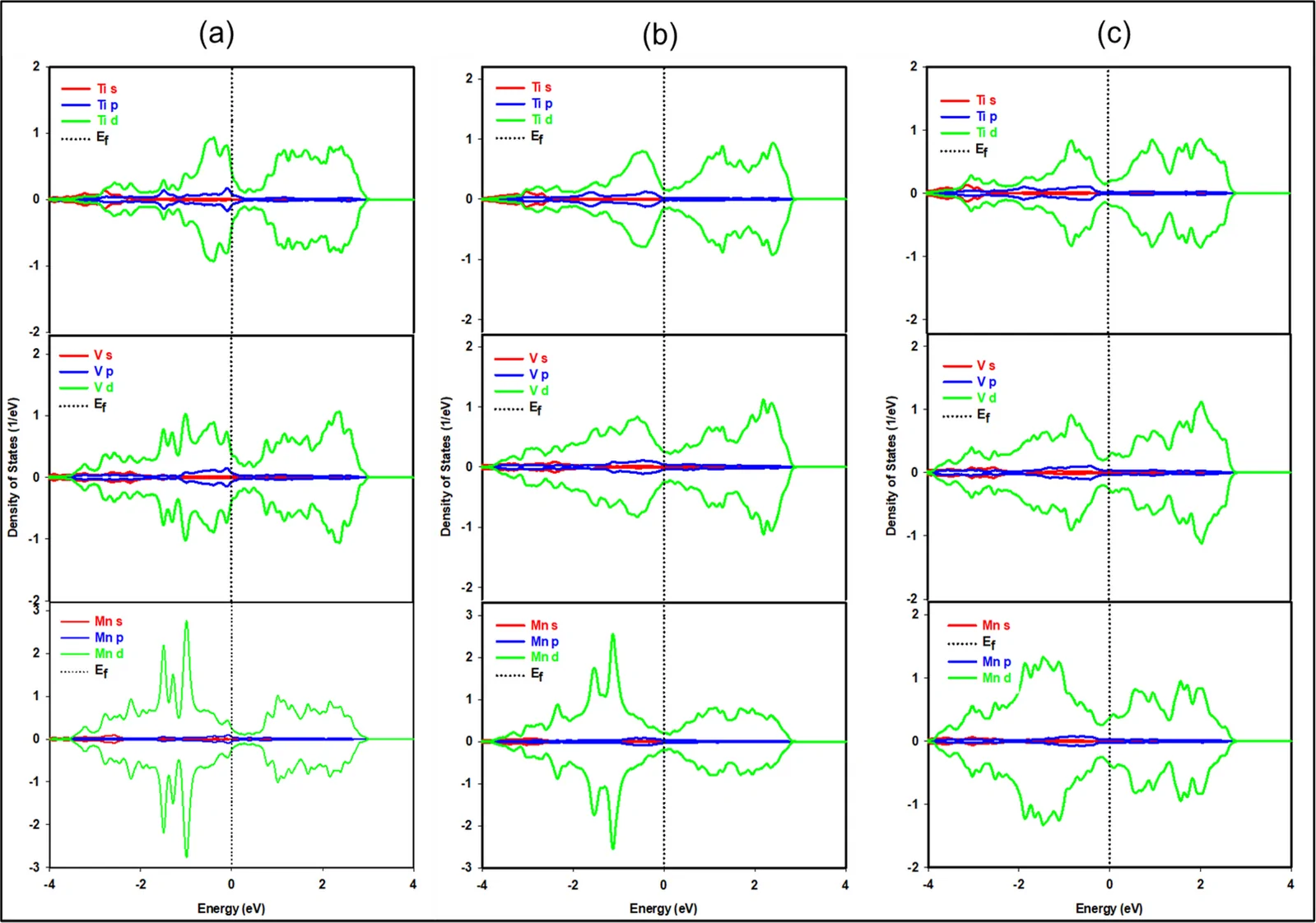
Partial density of states (PDOS) of the ground-state Ti–V–Mn alloy configurations: **a** Ti_4_V_6_Mn_2_, **b** Ti_8_V_9_Mn_7_, and **c** Ti_8_V_8_Mn_8_. The positive and negative DOS branches represent the spin-up and spin-down channels, respectively. The Fermi level is set at 0 eV

The electronic properties of the selected Ti–V–Mn ground-state configurations were investigated using partial density of states (PDOS) calculations to examine the orbital contributions governing the bonding characteristics and thermodynamic stability of the alloy system. The electronic structure calculations were performed using spin-polarised DFT. The calculated total magnetic moments were 0.0001, 0.0004, and 0.0001 μB for Ti_4_V_6_Mn_2_, Ti_8_V_9_Mn_7_, and Ti_8_V_8_Mn_8_, respectively, indicating negligible net magnetic moments for the investigated ground-state configurations. The calculated PDOS profiles for Ti_4_V_6_Mn_2_, Ti_8_V_9_Mn_7_, and Ti_8_V_8_Mn_8_ are presented in Fig. 3.

For all three configurations, the states around the Fermi level are dominated by Ti-3d, V-3d, and Mn-3d contributions, confirming their metallic character. The substantial overlap of these d-states indicates strong orbital mixing among the constituent transition metals. Differences between the configurations are most apparent in the distribution and intensity of the d-derived states. Ti_4_V_6_Mn_2_ exhibits sharper Mn-3d features in the occupied region, particularly between approximately − 2.5 and − 1.0 eV, indicating a comparatively more localised electronic contribution. In contrast, Ti_8_V_9_Mn_7_ shows broader and smoother d-state distributions near the Fermi level, consistent with greater electronic delocalisation. Ti_8_V_8_Mn_8_ displays an intermediate behaviour, with substantial d-state overlap and a relatively balanced distribution of Ti-, V-, and Mn-derived states around the Fermi level. These differences demonstrate that the calculated electronic structure varies with composition and atomic configuration, while all three structures retain metallic character.

**Fig. 4:**
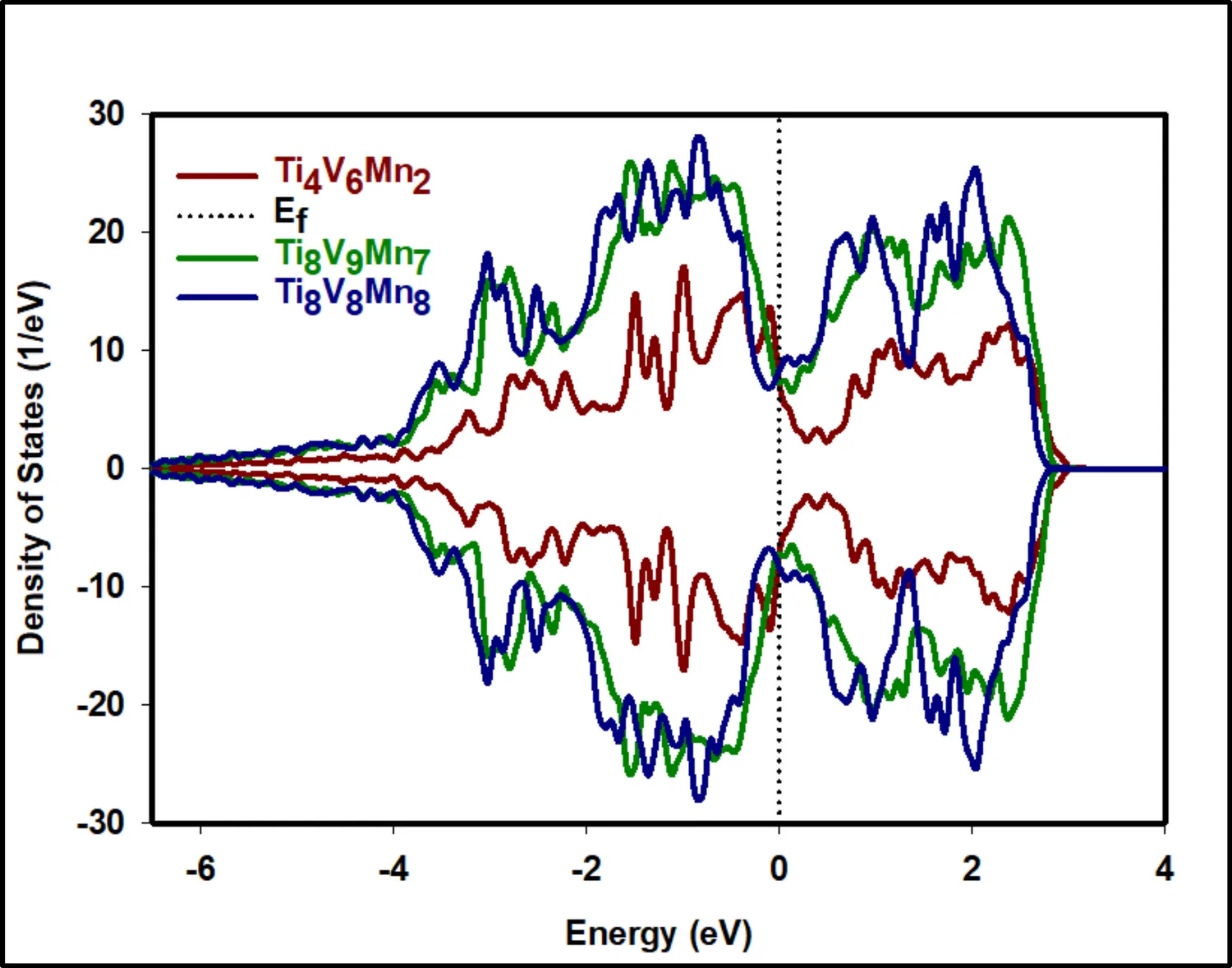
Total density of states (TDOS) of the ground-state Ti–V–Mn alloy configurations Ti_4_V_6_Mn_2_, Ti_8_V_9_Mn_7_, and Ti_8_V_8_Mn_8_. The positive and negative DOS branches represent the spin-up and spin-down channels, respectively. The Fermi level is set at 0 eV

The total density of states (TDOS) of the selected Ti–V–Mn ground-state configurations was calculated to further examine the overall electronic behaviour and metallic characteristics of the alloy system. The calculated TDOS profiles for Ti_4_V_6_Mn_2_, Ti_8_V_9_Mn_7_, and Ti_8_V_8_Mn_8_ are presented in Fig. 4.

It was observed that all investigated configurations exhibit finite electronic states at the Fermi level (*E*_f_ = 0 eV), confirming the metallic nature of the Ti–V–Mn alloy system. The absence of an energy band gap near the Fermi level indicates continuous electronic conductivity and significant electronic delocalisation within the alloy lattice, which is characteristic of transition metal–based alloys dominated by *d*-electron interactions [15]. Furthermore, noticeable differences in the DOS distribution among the three configurations indicate that changes in composition and atomic ordering strongly influence the electronic stability and bonding characteristics of the investigated alloys.

The Ti_4_V_6_Mn_2_ configuration exhibits comparatively lower DOS intensity near the Fermi level together with relatively sharp electronic features within both the occupied and unoccupied energy regions. The presence of these sharper peaks suggests increased electronic localisation and reduced orbital delocalisation relative to the other configurations. This behaviour is consistent with the structural distortion and reduced symmetry associated with the Ti_4_V_6_Mn_2_ structure, where local atomic ordering induces anisotropic electronic interactions within the alloy lattice. Similar localisation effects in transition metal systems have been associated with reduced electronic uniformity and composition-dependent bonding environments [32].

In contrast, the Ti_8_V_9_Mn_7_ configuration displays a broader and smoother DOS distribution near the Fermi level, indicating enhanced electronic delocalisation and stronger metallic bonding interactions throughout the alloy lattice. The broader electronic distribution suggests improved overlap among the transition metal *d*-states, leading to enhanced orbital hybridisation and stabilisation of the electronic structure. This behaviour is consistent with the comparatively lower heat of formation obtained for Ti_8_V_9_Mn_7_, suggesting that increased electronic delocalisation contributes significantly to its enhanced thermodynamic stability. Enhanced *d*-orbital hybridisation in transition metal alloys has previously been reported to promote stronger metallic bonding and improved structural stability [32, 33].

The Ti_8_V_8_Mn_8_ configuration similarly exhibits metallic behaviour with substantial electronic states distributed around the Fermi level. However, compared to Ti_8_V_9_Mn_7_, the DOS profile of Ti_8_V_8_Mn_8_ exhibits slightly higher peak intensities within both the occupied and unoccupied energy regions, indicating moderate electronic localisation associated with the near-equiatomic atomic arrangement. Nevertheless, the relatively continuous DOS distribution across the Fermi level indicates strong metallic bonding and good electronic stability within the structure. Overall, the TDOS results support the PDOS analysis and demonstrate that the thermodynamic stability of the investigated Ti–V–Mn alloys is strongly governed by the degree of electronic delocalisation and *d*-orbital hybridisation within the alloy lattice.

### Mechanical properties

#### Elastic constants and mechanical stability

The calculated elastic constants of the Ti_4_V_6_Mn_2_, Ti_8_V_9_Mn_7_, and Ti_8_V_8_Mn_8_ ground-state configurations are summarised in Table 2. The elastic constants provide important insight into the resistance of a material against external deformation and are widely used to evaluate the mechanical stability of crystalline solids [34]. The elastic stiffness tensors were evaluated using the strain–stress method, with the symmetry of each relaxed structure taken into account during the strained calculations. For Ti_8_V_8_Mn_8_ with Pnma symmetry, the symmetry-reduced tensor contains nine independent elastic constants. The small additional terms obtained from the numerical fitting are within the calculated uncertainties and are therefore not treated as independent elastic constants. For the lower-symmetry Pm Ti_8_V_9_Mn_7_ structure, the corresponding symmetry-allowed off-diagonal terms were retained The computed elastic constants fulfil the mechanical stability criteria for all compositions examined in this study, as confirmed by the positive eigenvalues of the elastic constant matrices obtained from the Born stability analysis [35].

**Table 2:** Calculated elastic constants C_*ij*_ (GPa) of the ground-state Ti–V–Mn alloy configurations obtained from first-principles calculations

Elastic constants (GPa)	Structures		
	Ti_4_V_6_Mn_2_	Ti_8_V_9_Mn_7_	Ti_8_V_8_Mn_8_
C_11_	241.94	286.95	292.52
C_12_	125.57	138.07	128.25
C_13_	126.53	131.18	147.44
C_14_	0.15	__	−0.02
C_15_	0.12	−1.67	0.43
C_16_	0.40	__	−0.11
C_22_	241.08	272.63	295.43
C_23_	126.29	142.28	139.35
C_24_	0.13	__	−0.03
C_25_	0.12	2.17	0.35
C_26_	0.39	__	−0.09
C_33_	217.22	284.72	279.81
C_35_	0.09	1.46	0.43
C_44_	32.45	50.39	64.11
C_46_	0.00	−0.28	0.00
C_55_	33.16	80.15	56.96
C_66_	58.20	50.51	76.34

Among the investigated structures, Ti_8_V_8_Mn_8_ exhibits the highest principal elastic constants, with calculated values of C_11_ = 292.52 GPa, C_22_ = 295.43 GPa, and C_33_ = 279.81 GPa, indicating strong resistance against axial deformation. The relatively large elastic constants suggest enhanced lattice rigidity and stronger interatomic bonding interactions within the orthorhombic Ti_8_V_8_Mn_8_ structure. Similarly, Ti_8_V_9_Mn_7_ exhibits comparatively high elastic constants, with C_11_ = 286.95 GPa, C_22_ = 272.63 GPa, and C_33_ = 284.72 GPa, indicating good mechanical stability and structural robustness. In contrast, the Ti_4_V_6_Mn_2_ configuration exhibits comparatively lower elastic constants, particularly for the shear components C_44_ = 32.45 GPa and C_55_ = 33.16 GPa, suggesting reduced resistance to shear deformation relative to the other configurations. The elastic constants reveal a composition-dependent trend, where increasing Mn content from the V-rich Ti_4_V_6_Mn_2_ configuration towards Ti_8_V_9_Mn_7_ and Ti_8_V_8_Mn_8_ results in enhanced resistance to elastic deformation. This suggests that a more balanced occupation of the B-sublattice by V and Mn atoms promotes improved lattice stiffness and mechanical stability.

The observed variation in the elastic behaviour among the three configurations can be associated with differences in atomic ordering and electronic bonding characteristics within the alloy lattice. The enhanced elastic stiffness observed for Ti_8_V_8_Mn_8_ and Ti_8_V_9_Mn_7_ is consistent with the stronger *d*-orbital hybridisation and enhanced electronic delocalisation identified in the PDOS and TDOS analyses. Stronger metallic bonding interactions generally improve resistance to elastic deformation and contribute to greater structural rigidity in transition metal alloys [36]. Conversely, the comparatively lower elastic stiffness of Ti_4_V_6_Mn_2_ may be attributed to the increased electronic localisation and local lattice distortion associated with its lower structural symmetry.

#### Elastic moduli

The calculated bulk modulus (*B*), shear modulus (*G*), and Young’s modulus (*E*) obtained using the Voigt–Reuss–Hill approximation [37] are presented in Table 3. The Vickers hardness (${H}_{v}$) was estimated using the empirical model proposed by Chen et al. [39], based on the calculated shear modulus and Pugh’s ratio. The Chen model is expressed as ${H}_{v}=2{\left({k}^{2}G\right)}^{0.585}-3$, where G is the shear modulus and k=G/B is the ratio of the shear modulus to the bulk modulus [38, 39].

**Table 3:** Calculated elastic moduli and ductility indicators of the ground-state Ti–V–Mn alloy configurations obtained using the Voigt–Reuss–Hill approximation

Structure	Bulk (*B*) (GPa)	Shear (*G*) (GPa)	Young’s (*E*) (GPa)	Poisson’s ratio (*ν*)	Pugh ratio (*B*/*G*)	Vickers hardness (*H*_*v*_) (GPa)
Ti_4_V_6_Mn_2_	161.68	44.71	122.80	0.373	3.62	1.11
Ti_8_V_9_Mn_7_	185.26	63.77	171.61	0.346	2.91	3.53
Ti_8_V_8_Mn_8_	188.64	69.05	184.63	0.337	2.73	4.35

These mechanical parameters provide further insight into the compressibility, rigidity, and overall mechanical response of the investigated Ti–V–Mn alloys [40]. Moreover, it was found that Ti_8_V_8_Mn_8_ exhibits the highest Hill bulk modulus of 188.64 GPa, indicating the greatest resistance to uniform compression. The relatively high bulk modulus suggests strong interatomic cohesion and enhanced lattice stability within the orthorhombic structure. Ti_8_V_9_Mn_7_ exhibits a comparable bulk modulus of 185.26 GPa, indicating similarly strong resistance to volumetric deformation. In contrast, Ti_4_V_6_Mn_2_ exhibits the lowest bulk modulus of 161.68 GPa, suggesting comparatively higher compressibility and weaker resistance to external pressure.

A similar trend is observed for the shear and Young’s moduli. Ti_8_V_8_Mn_8_ exhibits the highest shear modulus (*G* = 69.05 GPa) and Young’s modulus (*E* = 184.63 GPa), indicating superior rigidity and stiffness among the investigated configurations. Ti_8_V_9_Mn_7_ also demonstrates relatively high mechanical rigidity, with calculated values of *G* = 63.77 GPa and *E* = 171.61 GPa. In comparison, Ti_4_V_6_Mn_2_ displayed significantly lower values of *G* = 44.71 GPa and *E* = 122.80 GPa, indicating reduced resistance to shear deformation and lower overall stiffness. The reduced mechanical rigidity of Ti_4_V_6_Mn_2_ is consistent with its lower-symmetry structure and comparatively weaker electronic delocalisation observed in the electronic structure analysis.

To quantitatively assess the calculated elastic response, the present results were compared with first-principles results reported for closely related Ti-based C14 Laves phases. Ali et al. reported Voigt–Reuss–Hill bulk, shear, and Young’s moduli of 195, 71, and 190 GPa, respectively, for TiCr_2_ and 218, 64, and 174 GPa for TiMn_2_ [41]. The present Ti_8_V_9_Mn_7_ configuration gives corresponding values of 185.26, 63.77, and 171.61 GPa, which are within approximately 5–10% of the TiCr_2_ values and within approximately 15%, 1%, and 1% of the TiMn_2_ values for *B*, *G*, and *E*, respectively. Similarly, Ti_8_V_8_Mn_8_ yields 188.64, 69.05, and 184.63 GPa, differing from the TiCr_2_ values by only approximately 3% for each modulus. The lower values obtained for Ti_4_V_6_Mn_2_ are consistent with its comparatively lower elastic stiffness identified from the calculated elastic constants. Overall, the quantitative agreement with reported elastic properties of related Ti-based C14 Laves phases supports the physical plausibility of the calculated elastic response of the investigated Ti–V–Mn configurations.

The calculated Poisson’s ratios for all investigated configurations are greater than 0.33, while the corresponding Pugh’s ratios (*B*/*G*) are significantly larger than the critical value of 1.75 proposed by Pugh [42]. These results indicate that all investigated Ti–V–Mn ground-state configurations exhibit ductile mechanical behaviour. Ti_4_V_6_Mn_2_ exhibits the highest Pugh’s ratio equal to 3.62, suggesting enhanced ductility relative to the other configurations despite its comparatively lower elastic stiffness. The combination of relatively high bulk modulus and moderate shear resistance observed for Ti_8_V_8_Mn_8_ and Ti_8_V_9_Mn_7_ indicates that these structures possess a balanced combination of stiffness and ductility, which is desirable for structural hydrogen storage applications where resistance to brittle fracture is important.

The estimated Vickers hardness values also show a clear variation among the investigated configurations. Ti_8_V_8_Mn_8_ exhibits the highest calculated hardness of 4.35 GPa, followed by Ti_8_V_9_Mn_7_ at 3.53 GPa, whereas Ti_4_V_6_Mn_2_ has the lowest value of 1.11 GPa. The higher predicted hardness of Ti_8_V_8_Mn_8_ is consistent with its comparatively high shear modulus (69.05 GPa) and Young’s modulus (184.63 GPa), indicating greater resistance to plastic deformation within the empirical hardness model. Conversely, the lower hardness predicted for Ti_4_V_6_Mn_2_ is consistent with its lower shear modulus of 44.71 GPa. These values should be regarded as model-based estimates of hardness rather than direct experimental measurements.

## Conclusion

In this study, a combined CE and DFT investigation of AB_2_-type Ti–V–Mn alloys was performed successfully to examine the influence of configurational ordering on the structural, thermodynamic, electronic, and mechanical properties of the alloy system. The CE calculations successfully identified thermodynamically stable ground-state configurations from the convex hull, with Ti_8_V_9_Mn_7_ found to be more stable compared to Ti_8_V_8_Mn_8_ and Ti_4_V_6_Mn_2_ due to the lowest heat of formation. Structural analysis revealed that compositional variation significantly affects lattice symmetry, atomic ordering, and local coordination environments. Electronic structure calculations confirmed the metallic nature of all investigated configurations, with the bonding behaviour predominantly governed by Ti-3*d*, V-3*d*, and Mn-3*d* orbital interactions. Enhanced *d-*orbital hybridisation and electronic delocalisation were found to contribute to improved thermodynamic stability and stronger metallic bonding interactions. Mechanical property calculations further demonstrated that all investigated structures satisfy the mechanical stability criteria, while Ti_8_V_8_Mn_8_ exhibited the highest elastic stiffness and Ti_4_V_6_Mn_2_ displayed comparatively enhanced ductility. Overall, the results demonstrate that configurational ordering plays a critical role in governing the stability and structure–property relationships of Ti–V–Mn alloys.

These findings provide a computational foundation for future investigations of hydrogen absorption and hydrogen-induced embrittlement in Ti–V–Mn-based hydrogen storage alloys.

## Data Availability

Data reported in this paper are available upon request to the corresponding author.
